# Knowledge and comfort predict teaching about sexism in school teachers

**DOI:** 10.1007/s11218-025-10049-1

**Published:** 2025-04-29

**Authors:** Aífe Hopkins-Doyle, Lindsey Cameron, Lauren Spinner, Bridget Dibb, Andrea Kočiš, Rose Brett, Harriet R. Tenenbaum

**Affiliations:** 1 University of Surrey, Guildford, Surrey, GU2 7XH UK; 2 University of Kent, Canterbury, UK; 3 Education Endowment Fund, London, UK; 4 University of Portsmouth, Portsmouth, UK

**Keywords:** Sexism, School lessons, Teachers, Mixed methods

## Abstract

Although lessons about sexism can increase gender egalitarianism in children, teachers often shy away from discussing sensitive topics, such as sexism, in classrooms. We conducted two studies to examine why teachers may not discuss sexism. In a qualitative study with 20 primary school teachers, teachers reported not discussing sexism because of the belief that sexism was not an issue, low comfort, and low knowledge levels in teaching sexism, that sexism was less important than other topics, and not enough support from parents and schools. Teachers taught about sexism to balance out other perspectives, when they had support from authorities, and when sexism was related to a lesson. Using the themes found in Study 1, Study 2 developed quantitative measures to examine the predictors of intentions to teach about sexism among 233 primary and secondary school teachers. The full model found that teachers had higher intentions to teach about sexism when they felt more comfortable and knowledgeable about teaching sexism and when teachers were younger. We discuss findings from both studies in terms of theoretical and practical implications.

## Knowledge and comfort predict teaching about sexism in school teachers

According to the United Nations (2015), a sustainable future relies on gender equality (Goal 5, Sustainable Development Goals). Sexism, defined as stereotyping, prejudice, and discrimination based on gender, is a pressing barrier to achieving equality (Leaper & Spears Brown, 2015). In schools, children’s subject choices, academic beliefs, and achievement are impacted by gender stereotypes (Bian et al., 2017; Leaper, 2015; Starr et al., 2023). These stereotypes constrain behaviour and learning, and limit future educational and occupational aspirations (Leaper, 2015). Given that the goal of formal education is to create equitable opportunities for all (United Nations, 2015), schools are an ideal location for an intervention to combat gender inequality and sexism. Explicit lessons can reduce bias between social groups (Skinner & Meltzoff, 2019). Thus, school-based interventions can be used to reduce gender stereotyping (Aboud et al., 2012; Beelmann & Heinemann, 2014; Spinner et al., 2021), but teachers may not consistently employ interventions to reduce sexism. In two studies, the present research investigated whether teachers reported delivering lessons on sexism, which includes lessons on gender stereotypes, as well as what teachers perceived as the barriers and incentives on their intentions to teach sexism in the future.

Schools are often places where sexism and gender stereotypes are taught and reinforced through differential treatment of students (Gajda et al., 2022; Jovanovic & King, 1998; Kelly, 1988; Sadker & Sadker, 2010). Part of the reason may be that teachers themselves hold gender stereotypes or may not be aware of the stereotypical messages they communicate (Gajda et al., 2022). For example, when 7–8th grade teachers in Poland discussed gender with 13- to 14-year-old students in their school lessons, they tended to discuss historical sexism and role models (e.g. women as engineers) to combat gender stereotypes, but sometimes teachers would implicitly support gender stereotypes in their classroom lessons (Gajda et al., 2022). Indeed, to counteract gender stereotypes in the classroom, teachers encouraged children to act in gender non-conforming ways (e.g., asking the girls to be more ambitious) without acknowledging that some of these behaviours stem from gender stereotypes and roles, and that there are costs to acting in counter-stereotypical ways (Navarro et al., 2016).

Directly countering children’s stereotypes in the classroom by teaching about sexism is an effective means of reducing children’s gender stereotypes. In an intervention study, teachers counteracted children’s views about gender by teaching them to recognise sexism (Spinner et al., 2021). After four months of lessons, 6- to 10-year-old children felt more similar to the other gender and had more flexible beliefs about who could perform a range of gender-stereotyped occupations compared to a control group of students who did not learn about sexism. These findings suggest that lessons about sexism from teachers help to dispel beliefs about gender stereotypes.

Given the effectiveness of teaching about sexism, how frequently do teachers conduct lessons about sexism? Kaufman et al. (2024) compared US 3rd to 5th grade (children aged 8–11 years) teachers’ opinions about group discrimination based on gender, wealth, native language, religion, and race. Although teachers reported that wealth, native language, and race impacted educational outcomes, they did not believe that religion or gender impacted educational outcomes. Most teachers believed that discussing race (88%) and language (84%) was a good use of time with fewer believing that discussing religion (73%), wealth (66%), and gender (67%) was a good use of time. Following from their beliefs, teachers reported that they were most likely to discuss race and native language (Kaufman et al., 2024) than gender, wealth, or religion at least twice a term. Likewise, in a study looking solely at teachers’ beliefs about gender in Northern Ireland, 70% of primary school (children aged 4–11 years) teachers believed that they should challenge gender stereotypes in the classroom (Gray & Leith, 2004).

Less research has looked at *why* teachers may discuss sexism and gender stereotypes. Recently, a small qualitative study of 13 British Year 4–5 (children aged 8–10 years) teachers found that most believed that gender stereotypes were an important issue in primary school, that children in primary school were aware of stereotypes, and that children developed gender stereotypes in primary school (Gilchrist & Zhang, 2022). To our knowledge, there is no other research focused on understanding why teachers may choose or not choose to discuss sexism in the classroom.

Because of the limited evidence regarding the frequency and predictors of teaching lessons about sexism specifically, we draw on research about race/ethnicity-based discrimination to explore why teachers may choose or not to address sexism with students. Much of this research comes from the US. In one study, preschool (children aged 4–5 years) to second grade (children aged 7–8 years) teachers reported that they thought it was important to discuss race and felt comfortable doing so. At the same time, about 70% did not discuss race. Teachers argued that they chose not to discuss race because children were colourblind so it was not needed (despite their own reported evidence to the contrary), their own comfort levels discussing race, time, other topics that needed to be covered in the curriculum that have a higher priority than race, and the belief that it is parents’ responsibility to discuss race (Vittrup, 2016). Parents may play a central role in teachers’ decisions (Delale-O’Connor & Graham, 2019). Although more than 85% of pre-K (3–4 years) to high school (14–18 years) US pre-service and in-service teachers reported that it was important to discuss race, only about 30% thought that parents would be supportive.

Across two samples of kindergarten (aged 5) to high school (aged 18) US teachers, teachers had lower intentions to discuss race when they had higher implicit biases and were anxious of appearing racist (Tropp & Rucinski, 2022). However, reported school support was not related to decisions about whether to discuss race in the classroom. In contrast, Kaufman et al. (2024) found that higher school support, the belief that race affected education, and having a malleable theory of prejudice all influenced teachers’ decisions to discuss racism in the classroom. In sum, it seems that teachers’ comfort, concerns about time, teachers’ own views, and thinking it is their responsibility influences talk about race, whereas school support is a more equivocal predictor. Although we cannot generalise from race to gender, similar beliefs may underly teachers’ beliefs about discussing sexism in the classroom.

### The present studies

To understand why teachers may address sexism with students, we conducted two studies using an explorative sequential mixed methods design. Because of limited research, our first study was a qualitative investigation following a phenomenological epistemology with 20 teachers asking about their past experiences of teaching about sexism. We also asked teachers under what conditions they taught about sexism and the resources they would need to do so. Based on this study, we developed quantitative measures to predict teachers’ intentions to teach about sexism.

## Study 1

### Method

#### Participants

Participants consisted of 20 primary school teachers (5 men; 15 women) from England (*n* = 13) and Wales (*n* = 7) teaching in state schools recruited from social media and snowballing methods. Teachers taught children aged 4–11 years. We interviewed 20 teachers based on research (Guest et al., 2006) suggesting that 12–20 interviews are needed for saturation. Nineteen of these teachers were White British or White Other. We had some information about the diversity of 19 schools and the type of community in which the school was located for 17 schools from what teachers mentioned during the interviews. Nine teachers taught in ethnically diverse schools, and ten taught in schools with low diversity. The ethnically diverse schools tended to be composed primarily of children from Afghani, Indian, Pakistani, and Somali immigrant families who were Muslim. The teachers reported that the schools with low diversity tended to include about 10% of children from non-White British backgrounds. Ten teachers taught in suburban regions, five in a large city, and two in more rural areas.

#### Materials

We used one-to-one semi-structured interviews. Semi-structured interviews allow the opportunity to build rapport with the participant that facilitates conversation and allows detailed questioning and exploring of the topic. The interviews, guided by the interview schedule, explored whether teachers had taught lessons about sexism. The interviewer asked teachers to define gender stereotypes and sexism, whether they taught children about gender stereotypes and sexism, how they taught about these topics, where they obtained the resources to teach children about gender stereotypes and sexism, how children responded, what deters teachers from teaching these topics, and whether parents could be barriers to teaching these topics. The interview also included a discussion on race stereotypes and racism with children. However, this research presents only the gender stereotypes and sexism-related themes. The entire interview protocol may be found at https://osf.io/csyp4/?view_only=f84e0229a8314f429165d69aefd45a4e (see “original” tab).

#### Procedure

The University of Surrey provided ethical clearance for this study (FHMS 21–22 072 EGA). Prior to the main study, we piloted our interview script with two additional teachers (labelled Participant 1 and 2) and then modified the interview. Their data was not used. Participants were interviewed over MS Teams and received a £20 voucher. The interviews were conducted by the sixth author and lasted about an hour. Consent was gained at the time of the interview and the interview ended with a debriefing. All interviews were transcribed.

We analysed the transcripts using Thematic Analysis following the 6 steps suggested by (Clarke & Braun, 2021). The last and fifth author initially read through the transcripts and, using open coding, analysed the transcripts taking an inductive approach, which was then checked again by the last and fourth author in an iterative process. We clustered the codes into themes and subthemes.

### Results

Four themes were developed from the data: Theme 1 Barriers to Teaching Lessons on Sexism (with 7 subthemes), Theme 2 Reasons to Teach Lessons on Sexism (with 3 subthemes), Theme 3 Past Experiences Teaching Lessons on Sexism (with 4 subthemes), and Theme 4 Resources Teachers Needed (with 3 subthemes). Table 1 displays the themes and subthemes.

**Table 1:** Master table of themes

Theme	Subtheme
Theme 1: Barriers to Teaching Lessons on Sexism	1.1: ‘It’s not an issue’
	1.2: Teachers’ own views
	1.3: Teachers’ comfort
	1.4: Teachers’ knowledge
	1.5: Less important than other topics
	1.6: Giving in to parental pressure
	1.7: Roadblocks from schools
Theme 2: Reasons to Teach Lessons on Sexism	2.1 Need to balance out other opinions 2.2 Support of authority figures 2.3 Related to a lesson
Theme 3: Past Experiences Teaching Lessons on Sexism	3.1 Teaching about general stereotypes 3.2 Teaching about toys 3.3 Teaching about occupations 3.4 Teaching about domestic jobs and roles
Theme 4: Resources Teachers Needed	4.1 Video Recordings 4.2 Discussion questions 4.3 Vocabulary

#### Theme 1: barriers to teaching lessons on sexism

We developed this theme from the large number of comments that participants made in response to being asked what stops them from teaching sexism in class. Teachers identified a range of reasons why they would not teach lessons on sexism. We classified these reasons into seven subthemes. We describe each subtheme supported by quotes from the participants. Appendix A displays longer quotes as noted by an asterisk.

##### Subtheme 1.1: It’s not an issue

This subtheme showed that many teachers did not perceive sexism to be an important issue. Participants commented both on their own views and their perceptions of other teachers. For example, Participant 14 relayed, *“Because I don't want to make an issue of something when it isn't really an issue.”* Discussing other teachers, Participant 5 observed, *“I think for some people they don't really see it as an issue.”*

Two comments referred specifically to sexism not being an issue in primary school because of strategies put in place to ensure boys and girls are treated in the same way with similar opportunities. Sexism was perceived as more of an issue for secondary schools. For example, Participant 15 remarked, “*I think when it comes to sort of sexism and gender equality, I don't notice it to be too much of an issue in the primary school…”*.*

Thus, many participants did not view sexism as an issue that affected students in the primary years. Participants argued that they had not noticed sexism as a problem in schools and so concluded that it was not an issue. Teachers worried that if they were to teach about sexism, it would increase the salience of gender.

##### Subtheme 1.2: teachers’ own views

Teachers may not have perceived sexism as an issue because of their own beliefs. In this subtheme, participants made explicit comments that showed their beliefs and prejudices toward teaching sexism. In this subtheme, teachers expressed their own values and discussed their perceptions of the values held by their colleagues.

Participant 21, below, gave an example of how some staff refuse to engage in some school-wide inclusive behaviours due to their own prejudices, “*I've had one teacher at work that's extremely religious and we've got the pride.., the LGBTQIA* + flag—there's little pins, the management, we all wear them. And she wouldn't have one because she told me she hates gay people.”*

Other participants discussed their own views, such as Participant 15 who described not being aware of sexism having bothered anyone. For this, the teacher concluded that one should not over accommodate for sexism either, “not to go too far the other way either… not that it's ever been brought to my attention, it’s ever bothered any other children if you say right can I have the boys here and the girls there.”*

The quotes above suggest that there may be a range of reasons why the teachers thought that they should not address sexism, ranging from not being aware of its importance to being openly hostile to addressing these issues. This theme highlighted that teachers are also embedded in microsystems with specific values and beliefs.

##### Subtheme 1.3: teachers’ comfort

This subtheme was related to subtheme 1.2, teachers’ own views, and was mentioned by many teachers, who reported that they felt uncomfortable discussing sexism in the classroom. For example, Participant 20 spoke about how they considered sexism as a sensitive topic. Thus, they argued that teachers were fearful of bringing the subject up, “they're scared of saying something wrong.”* Other participants echoed this fear.

While one teacher spoke of the fear of offending, Participant 7 discussed teachers feeling restricted in what they feel able to do and say, as a result of their fear of saying the wrong thing *“teachers don't want to make a mistake and say the wrong thing and that can really inhibit what you do.”* Participant 21 reported that teachers may feel generally uncomfortable with the topic, “*they don't feel comfortable asking those questions*”. Thus, many teachers felt apprehensive about teaching about sexism.

##### Subtheme 1.4: teachers’ knowledge

Part of the reason that teachers may not have felt comfortable was because they judged that they were often lacking knowledge, including appropriate language, needed to discuss sexism. Indeed, many teachers mentioned that they lacked enough information and the appropriate vocabulary. Participant 15, for example, argued that they would feel uncomfortable because “*not using the correct language.”* These concerns were mentioned by several participants, for example, Participant 5 spoke of worries, *“I worried when I was teaching more sensitive issues about whether I was using the right terminology. …And yeah, and just generally feeling ill equipped.”*

Participant 10 revealed that they felt they did not know enough and would want more training first, *“I'm not informed enough about it as well. I think it could be that it's probably [a] lack of information on my part umm and I've never seen it really delivered before.”*

Participant 11 thought that they would more likely teach about sexism if they had better resources to help them with selecting appropriate vocabulary, “like a crib sheet for teachers.”*

##### Subtheme 1.5: less important than other topics

The first four subthemes refer more to internal than external factors. This subtheme could be internal or external in that teachers may personally think that maths and English are more important or that others (e.g., school administration) think that these subjects are more important. In this subtheme, participants suggested that even if sexism were something that should be taught, there were more important topics to address that took precedence over the teaching of sexism. For example, Participant 11 alluded to core subjects being more important than teaching sexism. Indeed, this teacher argued that the goal of school was to teach these subjects and not address sexism beliefs, “*really, it's (teaching sexism) not a massively high level of priority, you need to get your maths or English.*” Participant 17 similarly argued, “they just think that there are other things that are more important.”* Here teachers conceded that while sexism might operate in primary schools, other topics that needed to be covered took precedence. Teachers in our sample have a national curriculum that they need to follow, which may crowd out their ability to teach other topics.

##### Subtheme 1.6: giving in to parental pressure

The participants were also concerned about how parents might react to some of the lessons. Participant 5 supported the notion that parents could intimidate teachers from teaching sexism. As they said, “*a lot of teachers are very scared of parents and, like, parent backlash.*” Their views were often based on previous experiences, such as Participant 22, recounted a negative experience where parents objected and the teachers obliged, “Parents …said ‘I don't want my son to be exposed to this umm in our religion, this is disgusting’”* These quotes revealed that the views and behaviour of parents impacted on what teachers taught. Moreover, in some cases teachers were ‘scared’ of how they approached sessions on sexism and gender because of parents’ beliefs.

Some participants held the view that part of the reason some teachers were afraid of parents was based on what teachers perceived as the influence of cultural norms or religious beliefs in the families of the children they taught. Teachers did not always perceive that these groups shared liberal values about gender equality. Participant 13 exemplified this tension with an example of gender norms within some families in which mothers continued to not work outside the home after children had entered formal schooling. As she recounted, “*we kind of like try and promote like ohh ‘you don't have to just be like get married and be a mum as girls you should have dreams of your own’”.* She explained that communicating such messages was in contrast to what children saw in their families when, “*the majority of their mothers stay at home even once the children have gone to school, they don't work.*

The same participant also referred to their perception of the influence of religion on children’s gender stereotypes. She mentioned a child who would tell other children, “‘oh, they should stay at home, that they shouldn't be doing this, they shouldn't be doing that. You don't do this because otherwise you're going to hell.’”* Here the teacher discussed that the family’s gender stereotypes, rooted in religion, were repeated by a child in the class. These teachers tended to teach in ethnically diverse schools with many immigrant families. Given that the majority of the teachers were mostly White British, there may have been cultural differences between teachers and parents leading to a lack of communication.

##### Subtheme 1.7: roadblocks from schools

Despite the backlash from parents and parents’ views, teachers argued that if they had support from the schools, they felt they would be able to teach sexism in the classroom. However, many argued that school management went beyond not supporting them and instead obstructed teachers. Participant 13 explained that she believed that she was not able to teach this topic because her senior leadership team (SLT) insisted on reviewing anything teachers said about sexism or gender first, “ Our SLT have said to us, like, do not answer…and then you'd have to check with SLT what it [answer] is like.”* Participant 17 provided another example of how school management prevented the teaching of sexism in the classroom. This example showed that addressing this problem needs to go beyond the individual teacher and include the institution. As she recounted, “I'd planned a lesson on, umm, ah, gender identities discrimination… and the head teacher said, “Do not teach that lesson”.”* These two teachers among others explicitly discussed barriers to being able to teach about sexism in schools based on school management preventing this teaching.

#### Theme 2: reasons to teach lessons on sexism

We developed this theme based on comments where teachers mentioned why they had decided to teach about sexism in class. Teachers often discussed a need to counteract existing narratives, times when they received support from different authorities in children’s lives that made them believe that they should teach about sexism, and when they felt it was necessary because it was related to another topic. We discuss these themes in more detail with examples below.

##### Subtheme 2.1: need to balance out other opinions

Teachers reported concerns that children would learn inappropriately from media and parents. Teachers felt it was their duty to teach children so that their learning was informed. Participant 11, for example, explains their unease about children learning information from social media, “understand the world they live in, rather than just be swayed again by social media…generally teaching is about how we make critical minds.”* Participant 19 echoed these concerns with, “would you rather they went on the internet and found out a load of nonsense… would you rather be the appropriate adult who gives them guidance?”* In other words, teachers believed that they had a role to play in society.

Participants also spoke about the need to support and teach children when parents did not. Participant 9 described it as a ‘duty’ to challenge children’s beliefs. The teacher explained, “*Basically, a second parent, like, you have that duty to teach them that. Especially, I think, if their parents are against it…”.*

##### Subtheme 2.2: support of authority figures

In this subtheme, teachers mentioned that they taught lessons on sexism because of the support of parents and/or schools. As a result of this support, they felt comfortable teaching these lessons, which is a contrast to subtheme 1.7 in which teachers did not feel support from authority and did not teach about sexism.

For example, Participant 11 recounted a conversation with a parent where we can see how she felt positive in response to hearing she had support from the parent:

Like, I wasn't sure she was gonna say she's like, I love that. Like, I'm always talking about that with him. Like, she said, you talk about feminism too. And I was like, yes, literally all time, like, you're doing my job for me. Like, love that.

Participant 3 explained that her school would support her, “*As a school, I'd say we're all very kind of similar opinion that we want the children to understand what is right to say and what children … get upset by.”* And finally, Participant 10 knew that their, “*head will protect us very much about it very much*”. As a result of this support, this teacher was not afraid to teach about sexism.

##### Subtheme 2.3: related to a lesson

In this final subtheme, teachers relayed that they taught sexism because it was directly relevant to something they taught rather than being an additional lesson. Typically, these consisted of short parts of a lesson or comments during a lesson. For example, Participant 4 reported that she ensured she reminded students about historical changes in gender role when discussing the Anglo Saxons. She recounted that, “*women's job were to either have children or to sew. And the men's job was to go and … cut down trees and hunt, etcetera. And I always end it with, ‘But we know that's not the case now’.*”* Participant 7 similarly recounted how a history lesson evolved into a discussion about gender stereotypes, “*I’m teaching like history or something literally like talking about Romans. It probably was …And then we talked about stereotypes like that”.*

This participant continued, “I would address it and I and we have some really good conversations that come out of that because they are interested by it.”*

As Participant 6 mentioned, sexism was woven into lessons rather than a separate lesson. The participant recounted, “*So I think we touch upon it, but we don't call it necessarily sexism and we don't have a lesson about it. I think we just have it sort of woven in to what we do.”* In fact, the participant did not refer to sexism at all when describing the lesson.

#### Theme 3: past experiences teaching

In addition to teachers discussing reasons why they thought they should teach lessons on sexism, teachers also spoke about when they had taught about sexism in their classes. Teachers primarily had taught lessons that focused on broad stereotypes, and gender stereotypes in terms of toys, jobs, including domestic roles and manual jobs. Teachers tried to challenge children’s beliefs to demonstrate new ways of approaching these topics.

##### Subtheme 3.1: teaching about general stereotypes

Teachers often taught about general stereotypes to their students. They often spoke about having a debate and asking students if they agreed with stereotypes. In this theme, teachers did not always name the particular stereotypes in the interviews or named a few stereotypes.

Many, such as Participant 22, explicitly addressed the children’s stereotypes in what the teacher considered age-appropriate terms and led discussions across six lessons. The teacher explained to the children that a stereotype might be that, “*all girls like pink, all boys like blue… girls are gonna be more emotional, but boys aren't allowed to be emotional.”* The discussion focused on challenging these so that children understood that they did not have to follow these stereotypes.* Participant 13 similarly mentioned challenging stereotypes with the upper end of primary school. As they related, “*so it's basically about giving them stereotypes that they already have embedded and then challenging them.”*

Through their lessons, teachers tried to dispel children’s stereotypes. Teachers worried about the effects of not teaching children to challenge stereotypes early enough so that children’s understanding of how to disprove stereotypes was ‘embedded’. Participant 19 recounted, “So I feel like it needs to be taught younger… so they can start to embed it in their mind.”*

All these teachers believed that supporting children to challenge sexism and dispel stereotypes was critical in primary school. Subtheme 3.1 discussed here, included general stereotypes rather than more specific stereotypes, which is the focus of the remaining subthemes in this theme.

##### Subtheme 3.2: teaching about toys

Many teachers helped students understand that children should be allowed to play with any toy that they wanted rather than being restricted to toys stereotyped for a particular gender. For example, Participant 21 led a discussion with young children to help them understand that their gender did not limit the toys with which they could play, “all the other children were saying, Oh, no, but that you know, that's for girls.”* The teacher explained that she liked to play with dinosaurs to help the children understand that gender should not limit toy choices.

Participant 15 challenged stereotypes with actual toy catalogues that have started to challenge gender norms to make her points concrete to young children. As they reported “*you can also see in catalogues where you might have girls playing on the superheroes page or boys playing with the dolls.”* By using this example, children were able to see counter-stereotypical pictures. Participant 16 also used the real-life example of her daughter to help children challenge these stereotypes. She asked them who would play with different toys and then explained that her daughter liked to play with masculine-stereotyped toys to help them understand that children can play with a range of toys.* Many teachers used the tangible example of toys, central in children’s lives, to help them understand that they did not have to follow stereotypes.

##### Subtheme 3.3: teaching about occupations

Like toys, children may have stereotypes about occupations. Teachers mentioned a variety of methods from role play to discussion of role models to teach children to expand their occupational stereotypes.

With young children, teachers created materials for play that counteracted stereotypes about occupations. For example, Participant 8 explained that they used the role play area to challenge stereotypes, “it might be a shop or a house or like a, you know, GP, that kind of thing. I had, like, a mixture of, like races and genders … some nurses that were also not just like white women.”* Here the teacher discussed an intersectional approach to changing stereotypes with young children.

With older children, many teachers mentioned leading discussions with children to help them debate stereotypes about occupation. For example, Participant 17 recalled a time when they asked students to draw a doctor or a nurse and found that “*99% of the time”* children drew a white female for the nurse and a white male for the doctor. The participant recounted that these used the activity as a “a stimulus for our discussion point.”* In this example, the teacher employed children’s stereotypes as a springboard for discussion, which then acted as a mechanism for debate. In this way, the stereotypes originated from the children.

Another way that teachers tried to get children to see beyond stereotypes was using role models. Participant 16 mentioned, “*And then, you know, we've ensured that we have chosen that name, it is May Jamison, the first female black astronaut in space*.” By focusing on a particular person, teachers demonstrated to children that not all individuals followed the stereotype. Across occupations, teachers mentioned a variety of ways in which they supported children to think beyond stereotypes.

##### Subtheme 3.4: teaching about domestic jobs and roles

Related to occupations outside the home were the domestic roles and jobs that people often have within the family. Teachers helped children question what they typically saw in their families. In this subtheme, teachers again used concrete examples and drew on children’s funds of knowledge by having them examine what went on in their own families. For example, Participant 11 discussed a lesson based on children’s experiences getting the children to think about their own lives. They asked children, “Why does Mummy have to do the washing up? Why does Mummy have to do the cooking? … Is that something dad could do?”* The teacher was able to help the children realise that either parent could do these jobs, and it was only because of stereotyping that one parent was more likely than the other parent to engage in these tasks.

In two cases, such as this quote by Participant 5, teachers used examples of a range of families to challenge stereotypes. Sometimes the teachers felt a little wary of doing this, linking to the first theme where teachers were not always comfortable teaching sexism. The teacher recounted a time when they selected different pictures of families (i.e., some same-sex parents), but added, “But I do remember we were quite edgy about putting it out.”*

In this subtheme, teachers focused on children’s knowledge to try to change their stereotypes about roles in the family. Across this theme, teachers taught lessons about sexism to challenge children’s stereotypes to make them more open to a range of toys, occupations and roles.

#### Theme 4: resources teachers needed

We ended our interviews by asking teachers which resources they would want or need to help them to teach about sexism in the future. Many answers were idiosyncratic, but the resources broadly fell into three types of categories, which included video recordings, discussion questions, and vocabulary.

##### Subtheme 4.1: video recordings

Teachers believed that video recordings would help them explain information to children in an accessible way. Moreover, they argued that videos were less prevalent than other resources. As Participant 9 recounted, “*There definitely aren't videos out there, and I think there can be, but sometimes they're more focused on secondary school*.” They argued that it was important to have videos of real people that reflect what happens in the real world, rather than cartoons. For example, Participant 22 argued that children needed, “actual real people doing these videos and explaining how they feel.”* Participant 16 similarly wanted video recordings with “people they can relate to” and those that allow interaction, that would, “[have] space for discussion within it not just the teacher doing all the talking.”* This teacher wanted recordings that were interactive*.* The teachers emphasised that the videos should lead to discussion rather than creating a static resource. The need for resources to develop discussions came up in Subtheme 4.2.

##### Subtheme 4.2: discussion questions

In this subtheme, teachers mentioned the need for interactive resources and discussions to help them address sexism with children. Teachers frequently used constructive methods rather than didactic methods to work with children. As Participant 5 confirmed, *“Not tell them what to think, but find out what they think and they can kind of discuss it with each other. So question prompts would be really nice*.” Other teachers concurred with this viewpoint. Indeed, Participant 21 believed that it would be helpful to have, “*But yeah, having those those questions and having a big discussions always really, really helpful*”. Teachers wanted to engage children in discussions to help them understand this topic. Another example is Participant 20 who believed that discussion questions would help them. They argued, “*Either prompts like, easy, like child friendly ways to explain things. So I know how I would do it. But because I'm higher up in the school, I can go into more detail. And we can talk about more things because they're older*”.

##### Subtheme 4.3: vocabulary

This subtheme was related to *Subtheme 1.4 Teachers’ Knowledge*. Given that teachers felt that not having the appropriate knowledge, such as vocabulary, was a barrier, it follows that they would ask for such a resource. Participant 7 was explicit about the language that they would need. *“I think giving them [the children] some of that vocabulary that they can then access would be really good. So words like sexism and misogyny.”* Participant 20 echoed the need for children to have activities that helped them to learn the vocabulary, *“maybe, like, different age, or age appropriate definitions, potentially age appropriate things. And then I'd say just activities, activities to kind of embed it in them, because I don't feel like there's a lot..”*

Like subtheme 1.4, teachers voiced concerns that they did not know how to discuss the topic appropriately without this resource. Teachers felt, like Participant 4, that “*Because the generations have completely changed in terms of what's politically correct and what's not in terms of language.”* Teachers wanted children to learn, but did not want to make mistakes in what they taught the children.

### Discussion

This study examined teachers’ perceptions of why they had taught students about sexism. We coded four main themes in teachers’ interviews, which included barriers to teaching sexism in class, reasons to teach sexism in class, past topics teaching sexism, and what resources they would like to teach about sexism. Through in-depth, individual interviews with teachers, we were able to extend the research on ethnicity and race in teachers’ talk (Kaufman et al., 2024; Tropp & Rucinski, 2022) to understand in more detail when teachers choose to teach about sexism.

The first three themes involved discussions of barriers to and reasons for teaching sexism as well as when they had taught about sexism. The first theme focused on why teachers chose not to teach about sexism. Teachers mentioned that they did not believe sexism was an issue and often cited their own or other teachers’ views that they personally did not support ending sexism. They also spoke about their comfort and knowledge levels. Teachers were also concerned about fitting sexism into the topics that they needed to teach (that sexism was less important than other issues). Finally, many teachers were concerned about parents and school leadership. At the same time, teachers also noted reasons to discuss sexism in class. In the second theme, teachers reported that they discussed sexism when they believed that they should balance out other people’s opinions to expose children to different perspectives, when they had the support of authorities, and when sexism was related to a topic that they were teaching. Indeed, many teachers reported that they taught about sexism. In the third theme, teachers mentioned teaching about stereotypes, toys, occupations and domestic roles. Children often hold stereotypes about toy choices, which may constrain their activities and learning (Leaper, 2015). Children also frequently have gender-stereotyped interests (Weisgram et al., 2010), which may stem from their beliefs about who should perform some occupations (Liben et al., 2002).

Finally, we asked teachers about resources that they would like that would support them in teaching about sexism. Teachers mentioned that they believed that video resources, discussion questions, and vocabulary would be helpful. These resources are related to teachers’ concerns about lacking knowledge or feeling comfortable enough to teach about sexism. We found additional barriers to discussing sexism in the classroom than had been reported in quantitative research about discussing gender (Kaufman et al., 2024).

Although this study was able to develop a more in-depth understanding of why teachers discussed sexism as well as extend the research to understand how we can support teachers in these endeavours, it is not without limitations. This study included 20 teachers, so it is difficult to know if these reasons generalise beyond our sample especially because we mostly recruited through social media. Moreover, it was confined to primary school teachers. In fact, in the first subtheme (1.1: ‘It’s not an issue’), many teachers argued that teaching about sexism was something that belonged in secondary school rather than primary school because often young children were not aware of gender differences. We wanted to see, thus, whether secondary school teachers agreed with this viewpoint. Beyond sample constraints, there are also potential psychological beliefs that could influence teachers’ intentions to discuss sexism with children and influence the reasoning that emerged in our qualitative analysis. For example, research shows that people who endorse hostile and benevolent sexism are more likely to perceive the gender status quo as fair (Becker & Wright, 2011; Jost & Kay, 2005). However, the primary purpose of this qualitative study was to inform the design of Study 2 where we measure the main themes arising in Study 1 to determine their association with decisions to teach sexism.

## Study 2

Study 2 examined the perceived factors predicting teachers’ future intentions to teach sexism in the classroom. Study 1 focused on primary school teachers’ current behaviour and identified barriers and incentives to teaching sexism. Building upon Study 1, we developed questionnaire measures to quantitively assess intentions to teach sexism, and the barriers and incentives to addressing sexism in the classroom. In this way, we were able to extend the findings of Study 1 by including a large range of reasons that we had developed through our interviews with teachers. In addition, we investigated the potential role of teachers’ personal beliefs and ideologies, which were highlighted in Study 1, on intentions to teach about sexism. Indeed, research on whether teachers discuss racism finds that implicit bias predicts discussions (Tropp & Rucinski, 2022). For this reason, we examined the influence of teachers’ hostile and benevolent sexism (Glick & Fiske, 2018) and sexism socialisation (i.e., the belief that addressing sexism with children is important; Hilliard & Liben, 2020). Finally, we also extended our sample to include secondary as well as primary teachers. A broader sample allowed an assessment of the potential common and unique factors predicting intentions to teach about sexism across school levels.

Based on Study 1, we hypothesised that teachers’ intentions to teach sexism in the future would be predicted by (1) higher endorsement of the belief that sexism remains an issue, (2) higher sexism socialisation beliefs, (3) higher levels of comfort in teaching sexism, (4) higher levels of knowledge about sexism, (5) lower levels of parental pressure, (6) higher levels of school support, (7) higher levels of thinking teachers have a role to play in society, (8) higher levels of believing that the topic is relevant to lessons, (9) lower levels of thinking that the topic was too politically sensitive, and (10) lower levels of benevolent sexism, and (11) lower levels of hostile sexism. The hypotheses were pre-registered. We also explored the influence of teacher level (primary or secondary), age, and number of years of experience as well as controlled for teacher gender.

### Method

#### Participants

Participants comprised 233 current teachers (34 men, 199 women; 133 primary, 18 level unspecified, 82 secondary school). Teachers had taught for a mean of 13.54 years (*SD* = 7.72) and were 39.45 years old (*SD* = 9.18). A G*Power calculation (version 3.1) (Faul et al., 2007) calculated that to run a regression with 11 predictors at a power of 80% and alpha of 0.05 with a small to medium effect size (f = 0.08), a total of 221 participants was needed. To account for participants dropping out, an extra 10% were added which made a total of 243 participants needed. Our effect size estimate was based on Tropp and Rucinski (2022). They found that teachers’ intentions to discuss race was nonsignificant with school support, had a correlation of 0.16 with implicit bias, and a correlation of 0.48 with teachers’ confidence, which led us to estimate a small to medium effect size. However, there were only 233 British teachers who completed the study after one month on Prolific. One teacher incorrectly reported their age as 16, which we treated as missing and used listwise deletion for analyses involving age.

#### Materials

Teachers rated all items listed below from 1 (strongly disagree) to 5 (strongly agree), except Ambivalent Sexism. Intentions to teach sexism was our key outcome variable. All other variables were treated as predictors. Variables were keyed such that higher scores indicated a greater presence of that attribute. Some items were reverse scored. We created Intentions to Teach Sexism, Sexism Remains an Issue, Comfort Teaching Sexism, Knowledge about the Topic, Parents’ Pressure, School Support, Politically Sensitive, Role in Society, and Relevance based on Study 1. See Appendix B for details of exploratory factor analyses for these measures.

##### Intentions to teach sexism

Teachers rated their intentions to teach sexism in the future on four items (e.g., “I intend to discuss sexism with my class”) (α = 0.86).

##### Sexism remains an issue

Teachers rated the degree to which sexism remains an issue on four items (e.g., “Sexism is still a serious issue.”) (α = 0.77).

##### Sexism socialisation beliefs

We developed a measure based on Hilliard and Liben (2020). We assessed teachers’ beliefs that it is important to discuss sexism with children on seven items (e.g., “It is important to address sexism directly with children.”) (α = 0.89). The measure was originally created in respect of parents. We replaced “parents” in the items with “teachers”. We did not use the first item and final four items in the Hilliard and Liben scale to create a more targeted measure. We also added two items that were more similarly worded to the other items.

##### Comfort teaching sexism

Teachers rated their comfort levels teaching sexism on four items (e.g., “I fear I would say the wrong thing if I taught about sexism.”) (α = 0.74). Three items were reverse scored.

##### Knowledge about the topic

Teachers rated whether they perceived that they had enough knowledge about the topic on four items (e.g., “I am not familiar with the appropriate vocabulary for teaching lessons about sexism.”) (α = 0.86). All items were reverse scored so that higher scores indicated more knowledge.

##### Parental pressure

Teachers rated whether they perceived that parents supported the teaching of sexism on three items (e.g., “Parents would perceive teaching lessons about sexism as inappropriate.”) (α = 0.96). Higher endorsement indicates greater parental pressure to not teach about sexism. We removed a fourth item after conducting a factor analysis.

##### School support

Teachers rated whether they perceived that their school supported the teaching of sexism on four items (e.g., “My school leadership would back me if anyone complained that I taught about sexism.”) (α = 0.80). Two items were reverse scored.

##### Politically sensitive

Teachers rated their beliefs that the topic would be too politically sensitive to teach on three items (e.g., “Sexism is too political a topic to be taught in school.”) (α = 0.80). We removed a fourth item due to a low alpha (α = 0.69).

##### Role in society

Teachers rated whether they thought that teachers should play a role in society (e.g., “Teachers should play a role in making changes towards achieving gender equality.”) (α = 0.82). One item was reverse scored.

##### Relevance

Teachers rated whether they would discuss the topic if was relevant to what they were teaching on four items (e.g., “I would discuss sexism if it came up naturally.”) (α = 0.91).

##### Ambivalent sexism inventory (Glick & Fiske, 2018)

We assessed agreement with benevolent (e.g., “Women should be cherished and protected by men.”) (α = 0.79) and hostile (e.g., “Feminists are making unreasonable demands of men”) (α = 0.90) sexism with 6 items each (Rollero et al., 2014). Teachers rated their agreement with the statements from 0 (strongly disagree) to 5 (strongly agree).

### Procedure

We received ethical clearance from the Faculty of Health and Medical Sciences at the University of Surrey (FHMS 22–23 111 EGA). We pre-registered the study on the Open Science Framework (https://osf.io/csyp4/?view_only=f84e0229a8314f429165d69aefd45a4e) and uploaded the questionnaire and data to the website. However, we renamed two variables from our pre-registration. We renamed higher importance of teaching sexism to *sexism remains an issue* and less fear that parents will not approve to *parental pressure*. We also had planned that teacher gender would be an exploratory variable. Because of the small number of men, we entered teacher gender as a control rather than an exploratory variable. We recruited teachers on Prolific. Teachers took a mean of 10 min, 18 s (*SD* = 6 min, 2 s) to complete the questionnaire. After agreeing to participate, teachers completed information about their gender, which grade they taught, and their age. Next, they completed the measures described in the materials section. They ended by reporting whether they had ever taught about sexism previously and selected which types of materials from a list they would like created to teach about sexism.

### Results

#### Descriptive statistics

About half (*n* = 121, 51.9%) of the teachers reported having taught lessons on sexism previously. A one-sample *t*-test (against the mid-point) indicated that teachers were more likely to agree than disagree that they would discuss sexism in the future with children, *t*(232) = 8.76, *p* < 0.001, *d* = 0.83. Further independent samples* t*-tests for intentions to teach sexism in the future showed no differences by teacher gender, age (> or < median age = 38), or years teaching (> or < median years = 13), all *p*s > 0.09. Table 2 displays the materials teachers reported wanting. More than 50% of teachers selected real-life examples, slides, discussion prompts, videos, vocabulary, and ideas for role-play.

**Table 2:** Resources teachers would like

	Taught about sexism (*n* = 121)		Have not taught about Sexism (*n* = 112)	
	*N*	%	*N*	%
Real-life examples	96	79.34	81	72.32
Slides	94	77.68	79	70.54
Discussion prompts	92	76.03	82	73.21
Videos	85	70.25	77	68.75
Vocabulary	66	54.55	68	60.71
Role-play scenarios	51	42.15	56	50
Role models	50	41.32	40	35.71
Pictures	40	33.06	26	23.21
Lesson Plans	38	31.41	50	44.64
Guides	37	30.58	42	37.50
Websites with information	37	30.58	30	26.79
Worksheets	35	28.93	31	27.68
Books	30	24.79	23	20.54
Posters	23	19.01	25	22.32

#### Hypotheses testing

We conducted correlation analyses to examine relations between intentions to teach about sexism and the predictor variables. As expected, teachers’ intentions to teach sexism were related to higher endorsement of the belief that sexism is an issue, higher levels of sexism socialisation beliefs, higher levels of comfort, higher perceived knowledge, higher levels of school support, higher levels of wanting to play a role in society, and higher levels of relevance. Intentions to teach were related to lower levels of thinking that the topic was too politically sensitive as well as lower levels of benevolent and hostile sexism. Table 3 displays the correlations between all variables.

**Table 3:** Means and correlations of intentions to teach sexism

Variables	*M*	*SD*	1	2	3	4	5	6	7	8	9	10	11	12	13	14
1. Intentions to Teach	3.48	0.83	1													
2. Issue	4.00	0.62	0.48**	1												
3. Sexism Socialisation	4.00	0.62	0.63**	0.51**	1											
4. Comfort	3.69	0.78	0.56**	0.40**	0.51**	1										
5. Knowledge	2.95	0.94	0.44**	0.22**	0.33**	0.56**	1									
6. Parental Pressure	3.12	0.91	− 0.09	− 0.04	− 0.10	− 0.27**	− 0.31**	1								
7. School Support	3.31	0.82	0.31**	0.15*	0.30**	0.36**	0.41**	− 0.21**	1							
8. Politically Sensitive	1.75	0.74	− 0.48**	− 0.47**	− 0.57**	− 0.59**	− 0.34**	0.33*	− 0.20**	1						
9. Role in Society	4.16	0.64	0.55**	0.57**	0.65**	0.48**	0.20**	− 0.13*	0.16*	− 0.55**	1					
10. Relevance	4.36	0.58	0.50**	0.39**	0.60**	0.52**	0.32**	− 0.19**	0.19**	− 0.58*	0.54**	1				
11. Hostile Sexism	0.97	0.96	− 0.39**	− 0.51**	− 0.35**	− 0.35**	− 0.22**	0.13*	− 0.09	0.51**	− 0.49**	− 0.36**	1			
12. Benevolent Sexism	1.24	0.91	− 0.27**	− 0.33**	− 0.20**	− 0.24**	− 0.14*	0.18**	− 0.16*	0.39**	− 0.37**	− 0.31**	0.58**	1		
13. Gender	1.14	0.35	− 0.11	− 0.01	− 0.10	− 0.09	0.07	− 0.05	0.03	0.01	− 0.02	− 0.02	0.07	0.03	1	
14. Years Teaching	13.54	7.72	− 0.05	< 0.01	− 0.03	0.11	0.07	− 0.08	0.08	− 0.03	0.02	0.05	− 0.05	− 0.11	− 0.03	1
15. Age	39.55	9.07	− 0.12	− 0.02	− 0.06	− 0.11	0.04	− 0.12	0.09	− 0.04	< − 0.01	0.01	− 0.04	− 0.15*	− 0.04	0.75**

There are 199 women and 34 men in the sample.*p < 0.05; ** p < 0.01

To test our focal hypotheses regarding the predictors of teachers’ intentions to teach sexism (outcome variable) we ran regression analysis with all other variables as predictors. Age and years of teaching were also included as exploratory variables, and gender as a control variable. To maximise sample size, we did not include Teacher Level (primary or secondary teaching) because of the number of participants who did not specify level (*n* = 18). Instead, we carried out sub-sample analyses with available data for primary and secondary school teachers separately (see below). Before conducting the regression, we confirmed that there were no outliers using Cook’s distance and VIF (Field, 2018). The overall regression model was statistically significant, *F* (14, 215) = 19.02, *p* < 0.001. Table 4 displays full model statistics. When all variables were entered simultaneously, higher levels of sexism socialisation beliefs, higher levels of comfort, higher levels of knowledge, greater beliefs that teachers have a role to play in society, and being younger uniquely predicted intentions to teach sexism. There were no other significant predictors (all *p*s > 0.05).

**Table 4:** Regression coefficients predicting teachers’ intentions to teach sexism in class

	*B*	*SE*	*β*	*t*	*P*	95% CI B	
Constant	− 25	0.63		− 3.97	0.69	− 1.50	0.98
Issue	0.12	0.08	0.09	1.50	0.13	− 0.04	0.28
Sexism Socialisation	0.34	0.10	0.26	3.56	< 0.001	0.15	0.53
Comfort	0.17	0.07	0.16	2.32	0.02	0.03	0.32
Knowledge	0.17	0.05	0.19	3.18	0.002	0.06	0.27
Parental Pressure	0.08	0.05	0.08	1.62	0.11	− 0.02	0.17
School Support	0.07	0.05	0.07	1.31	0.19	− 0.04	0.18
Politically Sensitive	0.01	0.08	0.01	0.13	0.89	− 0.15	0.17
Role in Society	0.19	0.09	0.14	2.03	0.04	0.01	0.37
Relevance	0.08	0.09	0.06	0.92	0.38	− 0.10	0.26
Hostile Sexism	− 0.03	0.06	− 0.03	− 0.60	0.60	− 0.14	0.08
Benevolent Sexism	− 0.06	0.05	− 0.07	− 1.19	0.26	− 0.17	0.05
Gender	− 0.19	0.11	− 0.80	− 1.72	0.09	− 0.40	0.03
Years Teaching	0.01	0.01	0.05	0.70	0.48	− 0.01	0.02
Age	− 0.02	0.01	− 0.18	− 2.46	0.01	− 0.03	< − 0.01
*R*^2^	0.56						

Adjusted *R*^2^ = 0.52

To further explore potential differences in the factors influencing primary and secondary school teachers’ intentions to teach about sexism we ran sub-sample analyses. First, we conducted between-groups* t*-tests followed by correlation and regression analyses split by teacher level (primary vs secondary). Independent *t*-tests showed that secondary school teachers reported greater knowledge of sexism, *t*(213) = − 2.98, *p* = 0.003, *d* = 0.42, 95% CI [− 0.70, − 0.14], and primary school teachers reported greater perceived political sensitivity to teaching about sexism *t*(213) = 2.42, *p* = 0.016, *d* = 0.34, 95% CI [0.06, 0.62]. All other comparisons were non-significant (all *p*s > 0.08). The same pattern of correlations was found between intentions to teach about sexism and our predictors among both primary and secondary teachers. Tables 5 and 6 display these correlations. Follow-up regression analyses were conducted separately for primary and secondary teachers. Table 7 displays these statistics. For primary teachers, the overall model was significant *F*(14, 116) = 9.11, *p* < 0.001. Sexism socialisation beliefs, knowledge, and age were the only significant predictors. For secondary school teachers, the overall model was significant *F*(14,66) = 7.98, *p* < 0.001, but only sexism remains an issue and gender were significant predictors. Note that these regression models are underpowered and that the correlations with intentions to teach followed the same patterns in the subsample analyses for primary and secondary school teachers.

**Table 5:** Means and correlations of intentions to teach sexism in primary school teachers

Variables	*M*	*SD*	1	2	3	4	5	6	7	8	9	10	11	12	13	14
1. Intentions to Teach	3.43	0.78	1													
2. Issue	3.93	0.57	0.37**	1												
3. Sexism Socialisation	3.99	0.58	0.61**	0.45**	1											
4. Comfort	3.68	0.76	0.50**	0.33**	0.41**	1										
5. Knowledge	2.80	0.90	0.43**	0.15	0.29**	0.57**	1									
6. Parental Pressure	3.24	0.91	− 0.12	− 0.01	− 0.10	− 0.32**	− 0.43**	1								
7. School Support	3.35	0.77	0.28**	0.05	0.30**	0.36**	0.46**	− 0.26**	1							
8. Politically Sensitive	1.86	0.77	− 0.45**	− 0.41**	− 0.48**	− 0.54**	− 0.31**	0.38**	− 0.24**	1						
9. Role in Society	4.13	0.55	0.51**	0.55**	0.67**	0.40**	0.19*	− 0.13	0.10	− 0.51**	1					
10. Relevance	4.29	0.62	0.46**	0.31**	0.58**	0.50**	0.25**	− 0.17**	0.13**	− 0.50*	0.50**	1				
11. Hostile Sexism	1.05	0.93	− 0.34**	− 0.49**	− 0.32**	− 0.28**	− 0.23**	0.21**	− 0.03	0.49**	− 0.39**	− 0.29**	1			
12. Benevolent Sexism	1.32	0.91	− 0.19*	− 0.31**	− 0.17**	− 0.12	− 0.08	0.23**	− 0.08	0.38**	− 0.26**	− 0.26**	0.49**	1		
13. Gender	1.09	0.29	− 0.03	− 0.03	− 0.08	− 0.05	0.13	− 0.07	0.02	− 0.06	0.04	0.01	− 0.03	0.03	1	
14. Years Teaching	13.53	7.76	0.01	0.05	− 0.03	0.18*	0.10	− 0.08	− 0.02	0.02	0.08	0.01	− 0.11	− 0.13	− 0.02	1
15. Age	39.95	9.461	− 0.08	0.08	− 0.04	− 0.21*	0.09	− 0.16	0.02	− 0.07	0.11	0.04	− 0.10	− 0.21*	< − 0.01	0.73**

There are 121 women and 12 men in the sample. **p < 0.05; **p < 0.01

**Table 6:** Means and correlations of intentions to teach sexism in secondary school teachers

Variables	*M*	*SD*	1	2	3	4	5	6	7	8	9	10	11	12	13	14
1. Intentions to Teach	3.52	0.94	1													
2. Issue	4.06	0.69	0.59**	1												
3. Sexism Socialisation	4.00	0.68	0.64**	0.55**	1											
4. Comfort	3.70	0.82	0.63**	0.51**	0.62**	1										
5. Knowledge	3.18	0.96	0.42**	0.23**	0.32**	0.58**	1									
6. Parental Pressure	2.99	0.87	0.01	< 0.01	− 0.05	− 0.17	− 0.06	1								
7. School Support	3.23	0.91	0.31**	0.21	0.23*	0.32**	0.36**	− 0.16	1							
8. Politically Sensitive	1.61	0.70	− 0.52**	− 0.53**	− 0.73**	− 0.71**	− 0.33**	0.21	− 0.14	1						
9. Role in Society	4.18	0.76	0.58**	0.61**	0.61**	0.57**	0.16	− 0.06	0.17	− 0.64**	1					
10. Relevance	4.44	0.50	0.57**	0.51**	0.68**	0.62**	0.35**	− 0.07	0.29**	− 0.73**	0.64**	1				
11. Hostile Sexism	0.87	1.02	− 0.44**	− 0.55**	− 0.42**	− 0.47**	− 0.17	− 0.03	− 0.16	0.59**	− 0.64**	− 0.46**	1			
12. Benevolent Sexism	1.17	0.92	− 0.37**	− 0.35**	− 0.26*	− 0.39**	− 0.18	0.06	− 0.30**	0.39**	− 0.55**	− 0.38**	0.69**	1		
13. Gender	1.24	0.43	− 0.19	0.04	− 0.11	− 0.10	− 0.03	− 0.04	0.11	0.13	− 0.04	− 0.07	0.14	0.02	1	
14. Years Teaching	13.78	7.75	− 0.14	− 0.13	− 0.07	− 0.06	0.02	− 0.06	0.21	− 0.06	− 0.07	0.11	0.06	− 0.09	− 0.04	1
15. Age	39.16	8.40	− 0.21	− 0.17	− 0.14	− 0.09	− 0.03	− 0.06	0.16	0.02	− 0.19	− 0.04	0.07	− 0.07	− 0.04	0.82**

There are 62 women and 20 men in the sample. **p < 0.05; ** p < 0.01

**Table 7:** Regression coefficients predicting teachers’ intentions to teach sexism in class for primary school teachers (left-hand pane) and secondary school teachers (right-hand pane)

	Primary School Teachers (*n* = 132)							Secondary School Teachers (*n* = 82)						
	*B*	*SE*	*β*	*t*	*p*	95% CI B		*B*	*SE*	*β*	*t*	*p*	95% CI B	
Constant	0.16	0.82		0.20	0.84	− 1.45	1.78	− 1.73	1.56		− 1.11	0.27	− 4.84	1.38
Issue	− 0.01	0.12	− 0.01	− 0.08	0.94	− 0.24	0.22	0.33	0.15	0.24	2.24	0.03	0.04	0.62
Sexism Socialisation	0.39	0.14	0.29	2.79	0.01	0.11	0.66	0.36	0.18	0.26	2.02	0.05	< 0.01	0.72
Comfort	0.17	0.10	0.17	1.69	0.09	− 0.03	0.37	0.22	0.16	0.19	1.41	0.16	− 0.09	0.53
Knowledge	0.19	0.08	0.23	2.52	0.01	0.04	0.35	0.11	0.10	0.12	1.15	0.26	− 0.08	0.31
Parental Pressure	0.11	0.07	0.13	1.60	0.11	− 0.03	0.24	0.06	0.09	0.05	0.66	0.51	− 0.12	0.24
School Support	0.02	0.08	0.02	0.30	0.77	− 0.13	0.18	0.10	0.10	0.10	1.00	0.32	− 0.10	0.29
Politically Sensitive	− 0.07	0.10	− 0.07	− 0.74	0.46	− 0.27	0.12	0.19	0.22	0.14	0.86	0.39	− 0.24	0.62
Role in Society	0.21	0.14	0.15	1.54	0.13	− 0.06	0.49	0.18	0.17	0.15	1.09	0.28	− 0.15	0.52
Relevance	0.05	0.11	0.04	0.48	0.64	− 0.17	0.27	0.16	0.25	0.09	0.67	0.51	− 0.33	0.65
Hostile Sexism	− 0.05	0.07	− 0.06	− 0.68	0.50	− 0.19	0.09	0.09	0.13	0.10	0.74	0.46	− 0.16	0.35
Benevolent Sexism	− 0.05	0.07	− 0.06	− 0.69	0.49	− 0.18	0.09	− 0.12	0.12	− 0.12	− 1.02	0.31	− 0.37	0.12
Gender	− 0.09	0.18	− 0.03	− 0.50	0.62	− 0.03	0.27	− 0.39	0.17	− 0.18	− 2.28	0.03	− 0.74	− 0.05
Years Teaching	0.01	0.01	0.12	1.23	0.22	− 0.01	0.03	− 0.01	0.02	− 0.06	− 0.40	0.69	− 0.04	0.03
Age	− 0.02	0.01	− 0.23	− 2.34	0.02	− 0.07	< .− 01	− 0.01	0.02	− 0.08	− 0.54	0.59	− 0.04	0.02
*R*^2^	0.524							0.623						

#### Exploratory analyses

**Fig. 1:**
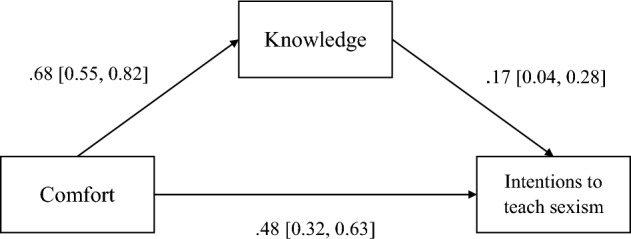
Analysis of indirect effects between comfort and intentions to teach sexism via knowledge. Coefficients and confidence intervals are bootstrapped (5000 resamples)

To explore the potential links between comfort and knowledge highlighted in Study 1, and in our correlational analyses, we conducted some additional exploratory analyses of indirect effects between intention to teach sexism (Y) and comfort levels (X), via knowledge (M) using the Process macro for SPSS (model 4, 5000 bootstrapped re-samples; v4.1, Hayes, 2018). The overall model was significant *F*(2, 230) = 57.24, *p* < 0.001, *R*^2^ = 0.33. There was a significant indirect effect via knowledge, *b* = 0.11, 95% *Bootstrapped* CI [0.03, 0.20]. Participants’ comfort levels significantly increased their knowledge which in turn increased their intentions to teach about sexism. See Fig. 1 for individual paths. Finally, given the differences in primary and secondary school teachers, we tested a moderated-mediation model. The model was the same as before with the addition of teacher level (primary or secondary) entered as a moderator (model 58, 5000 bootstrapped re-samples). There were no significant interactions on any of the individual paths (all *p*s > 0.06) but there was a conditional effect of the moderator on the indirect effect. For primary school teachers, the indirect effect of comfort on intentions via knowledge was significant, *b* = 0.12, 95% *Bootstrapped* CI [0.02, 0.23], whereas for secondary school teachers it was non-significant *b* = 0.05, 95% *Bootstrapped* CI [− 0.10, 0.24]. Given the small number of secondary school teachers in the sample these effects should be interpreted with caution.

### Discussion

Study 2 extended Study 1 by asking a larger sample of primary and secondary school teachers about their intentions to teach about sexism. In the correlational analyses, and largely consistent with expectations, teachers’ intentions to teach about sexism were related to higher endorsement of sexism socialisation beliefs, believing sexism remains an issue, higher levels of comfort and knowledge, higher levels of school support, higher levels of thinking that they have a role to play in society, higher levels of believing that the topic is relevant to lessons, lower levels of thinking that the topic was too politically sensitive, and lower levels of benevolent and hostile sexism. These findings were replicated in correlational analyses conducted separately for primary and secondary school teachers. When all predictors were entered in the model, teachers had higher intentions to teach about sexism in the future when they were high on sexism socialisation beliefs, when they felt more comfortable and knowledgeable about sexism, and when they were younger. We also conducted sub-sample regression analyses. We found that for primary school teachers, high sexism socialisation beliefs, higher knowledge, and being younger predicted intentions, but comfort followed the expected direction of effects. For secondary school teachers, believing that sexism remains an issue and being a woman predicted intentions. Higher sexism socialisation beliefs were a marginal predictor of intentions to teach about sexism in the future among secondary school teachers. We also asked teachers if they had ever taught about sexism; slightly more than half of the sample had taught about sexism in the classroom. We discuss these findings in relation to past literature.

Not surprisingly, teachers had higher intentions to teach about sexism when they had higher sexism-socialisation beliefs (the belief that teachers should prepare children for sexism) (Hilliard & Liben, 2020). In a study focused on US university students’ perspectives using this measure, 65% of young adults believed that parents should prepare children for sexism (Hilliard & Liben, 2020). Teachers in our study seem more likely to endorse the belief that they should discuss sexism than students did in Hilliard and Liben (2020). The different identity, teacher versus student, and their related levels of cultural experience might explain this difference. At the same time, in our study younger teachers were more likely to discuss sexism than older teachers suggesting that age is unlikely to explain differences between the present findings and Hilliard and Liben (2020). Future research may wish to explore an explanation for the difference.

Similar to Vittrup’s (2016) explanation of why teachers avoid discussions of race, teachers’ comfort levels predicted intentions to teach sexism. One reason may be that teachers reported that they did not have enough knowledge to teach about sexism. Higher comfort with sexism may support teachers in gaining additional knowledge. Exploratory indirect effects analysis showed that teachers’ greater comfort was associated with greater intentions to teach about sexism via increased knowledge about sexism. Further, moderated-mediation analyses indicated that this effect might be particularly important for primary school teachers, who reported less comfort than secondary school teachers (albeit replication with a larger sample of secondary teachers is needed). If teachers feel comfortable teaching sexism, they may seek out additional resources. Thus, creating resources, such as real-life examples, slides, and videos may increase the numbers of teachers willing to discuss sexism with children. Alternatively, knowledge may increase comfort, or they may have bi-directional influence. Longitudinal research may be able to confirm the direction of these effects.

Disconfirming our hypotheses, school support and parental pressure were unrelated to intentions to discuss sexism. Although school support sometimes predicted decisions to discuss race/ethnicity (Kaufman et al., 2024), it is not a consistent predictor (Tropp & Rucinski, 2022). Part of this reason may be that teachers in the UK may feel more protected in teaching about sexism because relationship and sex education is statutory as part of the National Curriculum in England (Department for Education, 2019). As a result, teachers may have felt less concern about parental pressure. Alternatively, being concerned about parental pressure may be confined to teaching children from particular backgrounds rather than a larger issue. Future research needs to examine how teachers’ concerns about parental pressure is related to school composition. This study suggests a range of reasons that may influence teachers’ decisions to discuss sexism with students.

## General discussion

Using qualitative and quantitative methods, we examined barriers and incentives in teachers’ intentions to teach about sexism. Study 1 enabled an in-depth analysis of why and how teachers decide to teach about sexism, an understudied area of research. Building on in-depth semi-structured interviews enabled us to generate and, in Study 2, test hypotheses about the importance of different factors in teachers’ decisions. Across both studies, the barriers centred on teachers’ lack of knowledge and comfort. Some of these reasons mirror why teachers do not discuss race. For example, teachers shied away from teaching about race when they did not feel comfortable (Delale-O’Connor & Graham, 2019; Vittrup, 2016). In Study 1, teachers did not think parents would approve of teachers discussing race (Delale-O’Connor & Graham, 2019). Specific to this study, the main facilitator was the belief that teachers should address sexism (sexism socialisation beliefs). We discuss these issues in relation to previous research and provide suggestions for future research and practice.

Awareness seems to be a central component of past teaching about sexism (Study 1) and intentions to teach about sexism (Study 2). In Study 1, awareness (i.e., Subthemes 1.1: It’s not an issue and 1.2: Teachers’ own views) was reported as a key perceived factor of whether participants had taught about sexism. Likewise, in Study 2, higher sexism socialisation beliefs and knowledge predicted intentions to teach about sexism. Sexism socialisation constitutes showing an awareness that sexism is a problem, that children need to be prepared to deal with it, and that it is a teachers’ role to address sexism and prepare students. People are often unaware that sexism continues to be an issue (Ashburn-Nardo & Karim, 2019). Some forms of sexism are easier to recognise as prejudice than others (Friesem & Levchak, 2019). For example, hostile sexism (e.g., overt discrimination and stereotypes) is easier to recognise than benevolent sexism (e.g., subtle views that may appear positive but reinforce gender inequality) (Hopkins-Doyle et al., 2019). In addition, these subtle forms of sexism, defined as hidden forms of unequal and unfair treatment based on gender, are less likely to be labelled as sexist (Friesem & Levchak, 2019). Further research is needed to examine the specific types of sexism teachers are familiar with and categorise as prejudice.

Another reason why sexism may be difficult to identify is the belief that boys and men may be disadvantaged (Anderson, 2015; Zehnter et al., 2021). Views about reverse sexism are becoming more prevalent and may inhibit awareness of the effects of sexism on girls and women. Our assumption is that teachers who recognised sexism were motivated to dispel its effects. Future research could ask teachers specifically about whether sexism continues to exist.

In addition, teachers who scored lower on hostile and benevolent sexism in the correlational but not regression analyses were more likely to report higher intentions to discuss sexism in the future. This finding mirrors research that shows lower implicit racial biases predict higher likelihood of discussions about race (Tropp & Rucinski, 2022). However, our findings suggest that when other factors are considered, hostile and benevolent sexism do not retain their predictive power. There may be important differences in how teachers approach discussions about sexism and racism, especially given that teachers believe that race affects educational outcomes more than gender (Kaufman et al., 2024).

In concert, the present studies also suggest that knowledge and comfort are key facilitators to teaching about sexism (Study 1) and intentions to teach about sexism (Study 2). The importance of knowledge and comfort were reflected in the resources teachers discussed across studies. In Study 1 (Theme 4), teachers highlighted videos, discussion prompts, and vocabulary, and this was consistent with teachers in Study 2 who endorsed these resources in additions to real-life examples as being most useful. The present findings are in line with previous work showing teachers felt uncomfortable teaching about sexism (Commeyras et al., 1997), and in a related intergroup context, race, teachers reported anxiety about appearing racist (Tropp & Rucinski, 2022). One possible explanation is that comfort could increase intentions to teach about sexism via greater knowledge. Exploratory findings of Study 2 lend some support to this assertion—showing that in the context of sexism, greater comfort increased intentions to address sexism with students via increased teacher knowledge. Further research is needed to replicate the indirect effect of knowledge, but this is a possible mechanism through which increased comfort can influence intentions to address sexism in the classroom.

There were some inconsistencies in the pattern of findings across studies. Some of the perceived issues mentioned in the qualitative study were not statistically significant when all factors were considered in the larger statistical model. However, that these factors did not predict intentions in the full model does not render them meaningless. Instead, it highlights that these issues are important for some teachers but not others. These differences may occur because some issues are less proximal for teachers at the aggregate level when different factors are considered at the same time. Future research could examine whether some issues prevent particular groups of teachers (e.g., urban, primary school teachers) from teaching about sexism.

### Limitations

In addition to differences between studies, we also found some differences between primary and secondary school teachers in Study 2. For example, primary school teachers reported greater perceived political sensitivity than did secondary school teachers, whereas secondary school teachers reported greater knowledge than primary school teachers did. There were also different patterns by level in the regression models, but not in the correlation analyses. Our sample was underpowered to conduct regression models separately by level of teaching, so these findings need to be interpreted with caution. Our study also used new measures to assess barriers and facilitators to teach about sexism. Factor analyses showed the expected four factor structure for measures developed from Themes 1 and 2 in Study 1, and all scales demonstrated very good reliability. However, given the small number of teachers and the heterogeneity of levels taught further research is needed to validate the questionnaire (Elson et al., 2023). Another limitation was that Study 1 only included primary school teachers. Future research needs to examine differences between primary and secondary school teachers’ views with a larger sample using mixed-methods approaches.

### Implications

Across both studies, *knowledge* was a central factor for intentions to teach about sexism. This finding points to a concrete implication with clear actions for practitioners, teachers, and principals. We recommend that preservice educators provide teachers with knowledge and resources through dedicated training. Evidence from Turkiye suggested that incorporating gender equity courses for preservice teachers was effective in increasing teachers’ desire to teach about sexism (Erden, 2009). Following from our findings, perhaps knowledge will increase teachers’ willingness to address sexism in the classroom.

On a positive note, personal ideological beliefs about hostile and benevolent sexism do not influence teachers’ intentions when other factors were considered. However, it should be noted that our sample of teachers in Study 2 scored very low on hostile sexism, and below the mid-point on benevolent sexism. Thus, among teachers with already low levels of sexism, our findings suggest that supporting teachers through knowledge-based training to teach about sexism may be effective. Further research is needed to examine how these processes might function among teachers with greater endorsement of ambivalent sexism.

### Conclusion

In sum, teachers’ intentions to teach about sexism were mainly predicted by their beliefs about their knowledge, comfort, and sexism socialisation beliefs across two studies. Pre-service and in-service training may be conducted to increase teachers’ knowledge about the topic, which may have the potential to increase teachers’ comfort and sexism socialisation beliefs. Increased comfort could also lead teachers to seek out additional knowledge creating a virtuous circle that would foster teachers’ willingness to teach about sexism. Given that explicit lessons about sexism increase children’s gender flexibility (Spinner et al., 2021), we need to support teachers to contribute to a more egalitarian and sustainable future.

## Figures and Tables

**Fig. 2:**
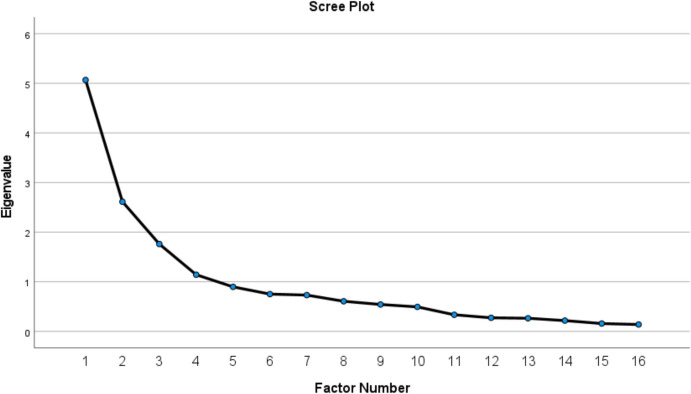
Scree plot for exploratory factor analysis of barriers to teaching lessons on sexism (N = 233)

**Fig. 3:**
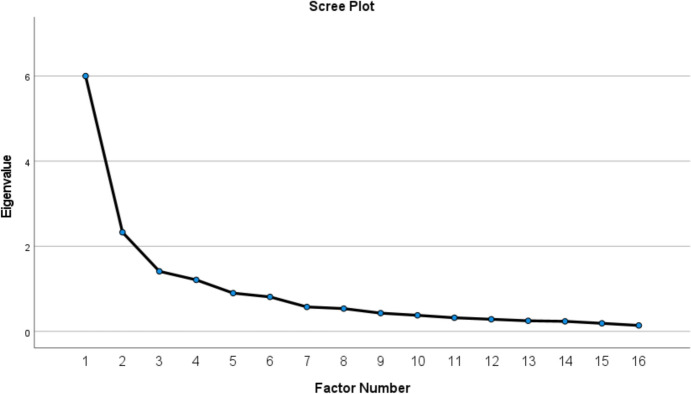
Scree plot for exploratory factor analysis of barriers to teaching lessons on sexism (N = 233)

## Appendix A

See Table

**Table 8:** Full quotes supporting themes in study 1

Theme	Subtheme	Quote
Theme 1: Barriers to Teaching Lessons on Sexism	1.1: ‘It’s not an issue’	Participant 15: *I think when it comes to sort of sexism and gender equality, I don't notice it to be too much of an issue in the primary school, whether other people with different experiences have had any issues with that. Umm, but from my point of view I don't seem to notice. I think maybe as children get older and that and they go into what comprehensive school that it might become more of an issue because I think generally in a primary school you generally treat the boys and girls the same any way. They have mixed lessons for PE and our sports club this year have changed from rather than having like boys’ football and girls’ football, although sometimes different um competitions outside of the school setting will say boys’ football team or girls’ football team. We've even gone now to like multi sports clubs and the boys and girls can both mix in those after school clubs as well*
	1.2: Teachers’ own views	Participant 21: *I would say, the core values as a person, you know, yes, we would like to think that all teachers are good people. But some people just aren't, you know, they might be the best teacher in the world, but actually core values wise… I've had one teacher at work that's extremely religious umm and we've got the pride. Well, the LGBTQIA* + *flag—there's little pins, the management, we all wear them. And she wouldn't have one because she told me she hates gay people* Participant 15: *But also I think, and I this is what my personal view probably is, that not to go too far the other way either, I think a lot of people are frightened out almost sort of say right ‘Can I have the boys here and the girls here’ because I suppose that as you get older there are gonna be children that don't want to identify as a boy or a girl and I think that's going to become a trickier issue as as sort of time goes on but umm I think in a primary school they, not that it's ever been brought to my attention, it’s ever bothered any other children if you say right can I have the boys here and the girls there*
	1.3: Teachers’ comfort	Participant 20: *But I know other people that are just, they see it as kind of taboo, or they're just like, oh, I don't have to answer tricky questions or things like that, and what's right and what's wrong, they're scared of saying something wrong. So I do get it from that point of view, you don't want to be teaching them the wrong thing, if you're not secure in your opinion of it*
	1.4: Teachers’ knowledge	Participant 11: *some of the language, sometimes, like a vocabulary bank, … so if you're gonna teach a lesson on gender stereotypes or, you know, LGBTQ plus type thing, you know, like, almost like a crib sheet for teachers*
	1.5: Less important than other topics	Participant 17: *I understand we're not there yet and it is a lot better, but you can't hierarchise these issues, and I think that's what some teachers try to do and I think going back to your earlier question where you said, you know, do all teachers have this view is sometimes not even necessarily that they disagree and they think it's not important, it's that a little bit like I said, they just think that there are other things that are more important*
	1.6: Giving in to parental pressure	Participant 22: *I'll give you a situation. Last year in my previous school, as I mentioned, ‘Julian wants to be a Mermaid’ is about a boy who sees mermaids and the undertone is that he might grow up to be transgender, he might be transgender, and he might grow up to transition. So a parent in the previous [year], this was, a Year 1 picture book, and they were studying it and writing, just so they could be, like, he likes, mm and just do simple sentences. Parents saw that they were studying it and kicked up, it like went to school, said ‘I don't want my son to be exposed to this umm in our religion, this is disgusting, blah, blah, blah’ … [a] teacher just gave in and went fine. We're not going to study it. So they pulled the book because it was just easier because that parent got another parent, another parent, another parent involved, and it became big. So they just went, I'm just going to stop it. Yeah* Participant 13: *And a lot of and we kind of like try and promote like ohh ‘you don't have to just be like get married and be a mum as girls you should have dreams of your own’ and that's really difficult when the majority of their mothers stay at home even once the children have gone to school they don't work* Participant 13: *and any stereotype or expectation that a [religious] person puts on females, for instance, like, ‘oh, they should stay at home, that they shouldn't be doing this, they shouldn't be doing that. You don't do this because otherwise you're going to hell.’ All of those things. He [the child] will just blurt out*
	1.7: Roadblocks from schools	Participant 13: *Our SLT have said to us, like, do not answer, you need to just say to the child, umm, ‘this is a good question. I'm gonna think about it and I'm going to come back and give you an answer’ and then you'd have to check with SLT what it is like* Participant 17: *I'd planned a lesson on, umm, ah, gender identities discrimination, that kind of thing. And I was working alongside the deputy head at the time. And she was planning with me. She knew what was going on. She knew that it was going to be age appropriate. But she must have had a conversation with the head teacher and the head teacher said, “Do not teach that lesson”*
Theme 2: Reasons to Teach Lessons on Sexism	2.1 Need to balance out other opinions	Participant 11: *umm understand the world they live in, rather than just be swayed again by social media. Social media, I suppose it's. Yeah. It's getting for me. It's about like, generally teaching is about how we make critical minds and questioning things and not accepting things as they are* Participant 9: “*Basically, a second parent, like, you have that duty to teach them that. Especially, I think, if their parents are against it because otherwise they're going home, they're hearing one message. We don't like that kind of people, that kind of person. Whereas if they come to school and hear something different, something that challenges that, it might open up their mind.”* Participant 19: *I said this earlier, like, would you rather they went on the internet and found out a load of nonsense. They don't know where to search, or would you rather be the appropriate adult who gives them guidance?”*
	2.2 Support of authority figures	Participant 3: *We've had to explain to children, like, if you say these things, it can actually come across like this. So, I think generally as a school, I'd say we're all very kind of similar opinion that we want the children to understand what is right to say and what children might, other people might, get upset by. So, I'd say we're all kind of very similar and kind of wanting them to be able to be aware of*
	2.3 Related to a lesson	Participant 4: *Sort of gender stereotype has been when we've been discussing history and then it's kind of, it kind of comes with the sense of sort of Anglo Saxons or something like that is where, you know, women's job were to either have children or to sew. And the men's job was to go and cut and hunt, cut down trees and hunt, etcetera. And I always end it with, ‘But we know that's not the case now’* Participant 7: *we haven't had, like explicit lessons about that. But like I said, because it's really important to me to sort of educate them about that, I do bring it in quite often. […] all of a sudden we got onto a tangent about gender stereotypes. And then I was like, wait, I’m teaching like history or something literally like talking about Romans. It probably was Romans, actually talking about Boudicca. I think then we talked about how, you know, women wouldn't have been seen as important enough to run like a tribe or whatever. And then we talked about stereotypes like that…* Participant 6: *We, I mean, I think we've covered it in regards to the fact that some of the people that we've discussed, so some of the sort of significant people we've said, you know, she, they were in a male dominated umm I think, I think one particular she was at a university where she was basically told she was a woman, she couldn't do it. So I think we touch upon it, but we don't call it necessarily sexism and we don't have a lesson about it. I think we just have it sort of woven in to what we do*
Theme 3: Past Experiences Teaching Lessons on Sexism	3.1 Teaching about general stereotypes	Participant 22: *so a gender stereotype might be that all girls like pink, all boys like blue, all girls are gonna do art and become mums. … just like, Oh, girls, girls are gonna be more emotional, but boys aren't allowed to be emotional. Those kinds of types. umm I would hope so because last half term we did six lessons on it. […]We do it very much in age appropriate terms. So it is very much like how I said it like oh, yeah, do you all girls like pink? And they're like, No, is it okay for boys pink? Yeah, you know?* Participant 13: “*The kids really got stereotypes by the time they get to Year 6, they're 10 and 11 now. … so it's basically about giving them stereotypes that they already have embedded and then challenging them, that's what we do in Year 6.”* Participant 19:* And it needs to be taught younger, I think they need to be aware of it younger, because the lessons have started to be very quick, because the children have very quick with the answers. And they know. So I feel like it needs to be taught younger, really, maybe Year 3 and 4, so they can start to embed it in their mind*
	3.2 Teaching about toys	Participant 21: *all the other children were saying, Oh, no, but that you know, that's for girls, we had the whole conversation of actually, you know, toys are toys. And this was in a reception class umm quite early on. So, they were quite little quite young, not really used to being at school. So yeah, we just had the conversation of, you know, toys are toys, it doesn't matter, you know, who you are what you are, because, you know, I sit you know, I like dinosaurs. But I'm a girl. Does that matter? A lot of no, no, of course, you know? Of course not. So why does it matter that you know, so and so wants to play with the Barbies? And they're oh it doesn't. Is it going to hurt them?* Participant 16: *You know, here's a card, here's a doll, who would play with each and actually unpicking it and saying, Is it okay? If a boy plays with a doll? And some of my boy-boys were like, No, they can't play with dolls that's girls. And I was like, whoa, whoa, whoa, hold on. And then I use my example, my daughter who loves playing with trains and cars and said is she's not allowed to play with that? And they're like, oh, no, she isn't like, so why is that okay, then?* Participant 8: *There were like more subtle ways I would try to kind of get it in. For example, when you always have a like role play area and every half term it might be something different, it might be a shop or a house or like a, you know, GP, that kind of thing. You know, I put all those ones in the bin and I tried to make some new ones that were like, obviously not just white people. And I think I tried to do like a couple pictures. So I had like. You know, like an Asian woman and like a black man or whatever is the doctor. And then I had, like, a mixture of, like races and genders for both, like, also, you know, some nurses that were also not just like white women*
	3.3 Teaching about occupations	Participant 17: *Yeah, we do. We do umm a lot of work on it really, one stand-out lesson that I can think of is we get the children, we give them blank sheets of paper and we say to them, for example, draw a doctor or a nurse, and 99% of the time for nurse, they'll draw a female who's white and for a doctor, they'll draw a white male. That's kind of a stimulus for our discussion point. And it's that kind of, I don't know what you call it in psychological terms, I want to say like, unconscious bias is it? Where you've already got that kind of preconceived idea of what you think it should be and I know that media doesn't really help or media didn't really help, I think it is getting a little bit better. But umm you know, even if you play that game with adults, nine times out of 10, they will do exactly the same*
	3.4 Teaching about domestic jobs and roles	Participant 11: *So we were able to debate well why? Why does Mummy have to do the washing up? Why does Mummy have to do the cooking? You know? Is that something dad could do? And generally they they were like well, yeah yeah they there's no reason why they couldn't. And so they they had chats about it and and gradually realized that most things you could shift to the either or but you know they’re anybody's job. Sort of stereotyping in terms of roles that they they did provided the basis for the lesson really and they they were able to see why actually just because it's done that way in our house doesn't mean that it has to be. So they're quite, they're quite useful lessons in that sense* Participant 5: *But I remember we consciously picked a picture or something that had like men and women doing all those different roles. And it also weaved them in with like what a family looks like and sort of how the different families can can look. And. But I do remember we were quite edgy about putting it out*
Theme 4: Resources Teachers Needed	4.1 Videos Recordings	Participant 22: *But then also, like, we don't grow up, we're not in a world full of cartoons. So, it'd be better to have actual real people doing these videos and explaining how they feel. Because at the moment, we have to make up what we think they feel like how would you feel, but we don't actually have a real human being explaining to us that has been through those situations, how they feel, and a lot of a lot of our things like about feeling umm to get our kids like at my level to get kids to understand. umm So, I think that would be something that I would be more interested in seeing for the kids* Participant 16: *umm videos, umm interactive videos, children absolutely love. umm So I think yeah, it'd have to be engaging. It'd have to be relevant and up to date. umm I think it would need time and space for discussion within it not just the teacher doing all the talking. umm I think maybe including other people they recognise or people they can relate to.”*

Not all subthemes include a longer quote so they are not included in this table

8.

## Appendix B

### Exploratory factor analysis for barriers and facilitators to teaching sexism measures in study 2

We ran two exploratory factor analyses (EFA; SPSS v 28.0.1) using maximum likelihood extraction with Promax oblique rotation. We chose to investigate scales related to each Theme separately due to the conceptual distinctions between themes and because we do not have sufficient power to estimate an EFA with 32 items (Pett et al., 2003). Eigenvalues greater than 1 were used to interpret the numbers of factors. For ease of interpretation factor loadings are from the pattern matrix (see Tables

**Table 9:** Pattern factor loadings for exploratory factor analysis of barriers to teaching lessons on sexism

Items	Factor			
	1	2	3	4
I do not feel like I have enough knowledge to teach lessons about sexism.*	**0.92**	0.03	− 0.02	0.00
I do not feel experienced enough to teach lessons about sexism.*	**0.89**	0.08	− 0.06	0.07
I am not familiar with the appropriate vocabulary for teaching lessons about sexism.*	**0.72**	− 0.04	0.07	0.03
I do not have training in teaching lessons about sexism.*	**0.62**	− 0.09	0.01	− 0.12
I fear I would say the wrong thing if I taught about sexism.*	0.35	− 0.20	0.08	0.30
Parents might complain if teachers taught about sexism	0.01	**0.93**	0.10	− 0.02
There might be a backlash from parents if lessons about sexism were taught in schools	− 0.01	**0.92**	0.02	0.02
Parents would perceive teaching lessons about sexism as inappropriate	0.02	**0.80**	− 0.11	0.05
Because addressing sexism with children is difficult, parents might want to defer to teachers’ expertise	− 0.20	0.25	0.03	0.08
Sexism is still a serious issue	0.02	− 0.01	**0.98**	− 0.11
Sexism has a lot of negative consequences and therefore needs to be addressed	− 0.02	0.01	**0.83**	− 0.01
Sexism is not an issue in modern societies.*	− 0.03	0.07	**0.52**	0.19
There are a lot more important issues than sexism.*	0.03	− 0.05	**0.36**	0.12
I would feel uncomfortable teaching lessons about sexism in my class.*	− 0.08	0.02	0.02	**1.02**
I would feel uncomfortable talking about sexism with children of different gender than mine.*	0.01	− 0.06	0.06	**0.61**
I would feel comfortable to teach lessons about sexism at a primary-school level	0.05	0.13	− 0.01	**0.45**

*Indicates reverse scored item. Factor loadings are in bold. Extraction method is maximum likelihood. Rotation method is Promax

9,

**Table 10:** Pattern factor loadings for exploratory factor analysis of reasons to teach lessons on sexism

Items	Factor			
	1	2	3	4
I would discuss sexism if it came up naturally	**0.95**	− 0.03	0.04	0.06
I would discuss sexism if a child asked a question about it	**0.89**	0.00	− 0.04	− 0.05
If we were talking about something and I needed to address sexism, I would	**0.81**	0.03	− 0.01	− 0.05
I would discuss sexism if it was directly related to a topic I was teaching (e.g., history)	**0.70**	0.04	0.00	− 0.01
As a teacher, I need to make a difference in creating gender equality	− 0.04	**0.90**	− 0.03	0.05
Teachers should play a role in making changes towards achieving gender equality	− 0.01	**0.86**	0.03	0.00
As a teacher, I should have an active role in combating sexism through my teaching	0.09	**0.85**	0.04	0.09
There is no adequate support from the school leadership in our school to teach lessons about sexism.*	− 0.06	0.00	**0.78**	− 0.08
Our school provides strong support for teaching lessons about sexism	− 0.02	0.06	**0.71**	0.16
Our school leadership would not support me to develop lessons on sexism.*	0.09	− 0.05	**0.66**	− 0.14
My school leadership would back me if anyone complained that I taught about sexism	0.00	0.02	**0.65**	− 0.04
Sexism is too political a topic to be taught in school	0.03	− 0.03	0.02	**0.88**
Sexism is too sensitive a topic to be taught in school	− 0.06	− 0.11	0.04	**0.80**
Children should not be taught about sexism because people have different opinions about the topic	− 0.04	0.01	− 0.02	**0.56**
Teaching about social changes related to gender issues would be a misuse of my position as a teacher.*	− 0.01	**0.24**	− 0.01	− 0.35
Teaching lessons about sexism could be very challenging if there are children who have different opinions about how men and women should be	0.00	0.20	− 0.08	0.35

*Indicates reverse scored item. Factor loadings are in bold. Extraction method is maximum likelihood. Rotation method is Promax

10).

#### EFA Theme 1 Barriers to Teaching lessons on sexism

In the first EFA, scales measuring subthemes from Theme 1 in Study 1 (Barriers to Teaching Lessons on Sexism) were entered (i.e., Sexism Remains an Issue, Comfort Teaching Sexism, Knowledge about the Topic, Parents’ Pressure). As expected, investigation of the scree plot (see Fig. 2), eigenvalues, and factor loadings (see Table 9) indicated four factors. Factor 1 was Knowledge about the Topic (factor loadings between 0.62 and 0.92). Factor 2 was Parents’ Pressure (factor loadings between 0.80 and 0.93). Factor 3 was Sexism Remains an Issue (factor loadings between 0.36 and 0.98). Factor 4 was Comfort Teaching Sexism (factor loadings between 0.45 and 1.02). One item (“I fear I would say the wrong thing if I taught about sexism.”) loaded to a similar degree across two factors—Factor 1 and Factor 4. Conceptually this item was designed to capture Factor 4 Comfort Teaching Sexism and given the very good internal consistency with other Comfort Teaching Sexism items (α = 0.74) were kept this as a four-item scale. A further item (“Because addressing sexism with children is difficult, parents might want to defer to teachers’ expertise”) did not load on any factor (all < 0.25). This item was designed to capture Factor 2 Parents’ Pressure but given its low factor loadings and the increase in internal consistency from the four item (α = 0.80) to three item scale (α = 0.90) we decided to drop it.

#### EFA theme 2 reasons to teach lessons on sexism

In the second EFA, scales measuring subthemes related to Theme 2 in Study 1 (Reasons to Teach Lessons on sexism) were entered (i.e. School Support, Role in Society, Politically Sensitive, Relevance). As expected, investigation of the scree plot (see Fig. 3) and factor loadings (see Table 10) indicated four factors. The eigenvalue for one factor (Factor 4) was slightly lower than others at 0.84, however it still explained 5.23% of variance so it was retained. Factor 1 was Relevance (factor loadings between 0.70 and 0.95). Factor 2 was Role in Society (factor loadings between 0.24 and 0.90). Factor 3 was School Support (factor loadings between 0.65 and 0.78). Factor 4 was Politically Sensitive (factor loadings between 0.35 and 0.88). One item (“Teaching about social changes related to gender issues would be a misuse of my position as a teacher”) loaded to a similar degree across two factors—Factor 2 (Role in Society) and Factor 4 (Politically Sensitive). Conceptually this item was designed to capture Factor 2 and given the very good internal consistency with other Role in Society items (α = 0.82) we kept this as a four-item scale. A further item (“Teaching lessons about sexism could be very challenging if there are children who have different opinions about how men and women should be”) loaded at 0.35 on Factor 4 Politically Sensitive but we decided to drop this item given poor internal consistency of the four-item scale (α = 0.69), compared to the three-item scale (α = 0.80).
